# COVID-19 and maintenance hemodialysis: a systematic scoping review of practice guidelines

**DOI:** 10.1186/s12882-020-02143-7

**Published:** 2020-11-10

**Authors:** Hossein Akbarialiabad, Shahin Kavousi, Aria Ghahramani, Bahar Bastani, Nasrollah Ghahramani

**Affiliations:** 1 Shiraz University of Medical Sciences, Shiraz Medical School, Zand Street, Shiraz, 7134845794 Iran; 2 Pennsylvania State University College of Medicine, 500 University Drive, Hershey, PA 17033 USA; 3 Medicine-Nephrology, Saint Louis University School of Medicine, 3635 Vista Ave, St Louis, MO 63110 USA; 4 Medicine and Public Health Sciences, Pennsylvania State University College of Medicine, 500 University Drive, Hershey, PA 17033 USA

**Keywords:** COVID-19, SARS-COV-2, RRT, Dialysis, ESRD

## Abstract

**Background:**

Coronavirus Disease 2019 (COVID-19) has substantially impacted the provision of medical services. During the pandemic, many medical services, including facilities providing care to patients with end stage renal disease faced challenges in safeguarding patients and staff while providing clinical care. This study aims to identify the extent, range, and nature of articles related to COVID-19 and maintenance hemodialysis to understand the research gaps and propose recommendations for future research.

**Methods:**

Using the terms: “Dialysis” OR “RRT” OR “Renal replacement therapy” AND “SARS-COV-2” OR “COVID-19” OR “novel coronavirus” OR “2019-nCov”, we performed a multi-step systematic search of the literature in the English language in Pubmed, Scopus, Embase, and Web of Science published from December 1, 2019, to May 13, 2020. Two authors separately screened the title and abstracts of the documents and ruled out irrelevant articles. We obtained a full report of the papers that met our inclusion criteria and screened the full texts. We conducted a descriptive analysis of the characteristics of the included articles and performed a narrative synthesis of the results. We conducted this scoping review in accordance with the PRISMA-ScR Checklist.

**Results:**

We included 22 articles in this scoping review. Perspectives (*n* = 9), editorials (*n* = 4), and case series (*n* = 5) were the most common types of articles. Most articles were from Italy and the United States. Seventeen (77.3%) of the articles focused on the topic of recommendation for outpatient hemodialysis units. While many of the recommendations overlapped in several articles, there were also many unique recommendations.

**Conclusions:**

most of the articles are based on single-center experience, which spontaneously developed best practices. Many of these practices have formed the basis for policies and guidelines that will guide future prevention of infection and management of patients with End Stage Renal Disease (ESRD) and COVID-19.

## Background

In late 2019, a cluster of patients with respiratory complaints was admitted to hospitals in Wuhan, Hubei province, China [1]. In one patient, the results of bronchoalveolar lavage and high throughput genome sequencing revealed a new virus of the Coronaviridae family [2]. The novel virus was named severe acute respiratory syndrome coronavirus (SARS-COV-2). This family was the causative agent of Severe Acute Respiratory Syndrome (SARS) in 2003 and Middle East Respiratory Syndrome (MERS) in 2012 [3]. The name “SARS-COV-2” was changed to Coronavirus Disease 2019 (COVID-19). During the next few weeks, this disease spread worldwide, and the World Health Organization (WHO) declared a pandemic state on March 11, 2020.

The median incubation period is 5.2 days [4]. The disease ranges in severity from mild symptoms in most cases to a harsh and deadly course [5, 6]. The majority of symptoms are constitutional and involve the respiratory tracts, but cardiac, renal, neurological, and gastrointestinal manifestations of COVID-19 have also been reported [6–9].

Due to the high burden of comorbidities, and their impaired immune response, patients with ESRD are more likely to develop severe complications of coronavirus disease [10, 11]. Global quarantine and social distancing measures aim to mitigate further spread of the disease [12], but they are not feasible for patients receiving in-center hemodialysis, or for the facilities providing dialysis care [6]. These patients require regular sessions that involve close interaction with staff in a limited physical space which increases the possibility of spread of infectious diseases [11]. Furthermore, many patients need to be repeatedly treated at the same dialysis area and require transportation, frequently public and shared, several times per week [13]. Given sub-optimal immune status, older age, and comorbidities of dialysis patients, dialysis units may be potential centers for the rapid spread of COVID-19 [11, 14–16].

Although there are numerous publications relating to dialysis and COVID-19, many are in the form of preliminary reports on small number of patients from single centers. The relative paucity of reliable available information about the infection among dialysis units and dialysis patients [13] presents a challenge in obtaining the most useful information that would help patient care efforts. We conducted a scoping review to identify the extent, range, and nature of articles related to COVID-19 and outpatient maintenance hemodialysis that have been published as of May 13, 2020, and to identify research gaps and to propose recommendations for future research.

## Methods

### Review questions

This systematic review was based on the recommendations of the Preferred Reporting Items for Systematic Reviews and Meta-Analyses (PRISMA) statement.

We attempted to address the following questions in the era of COVID-19:

- How can we provide a safe environment to prevent the spread of COVID-19 in the chronic outpatient hemodialysis unit?
- How can outpatient hemodialysis center administrators improve protocols to optimize staff performance and patient satisfaction?

### Search strategy

**Fig. 1:**
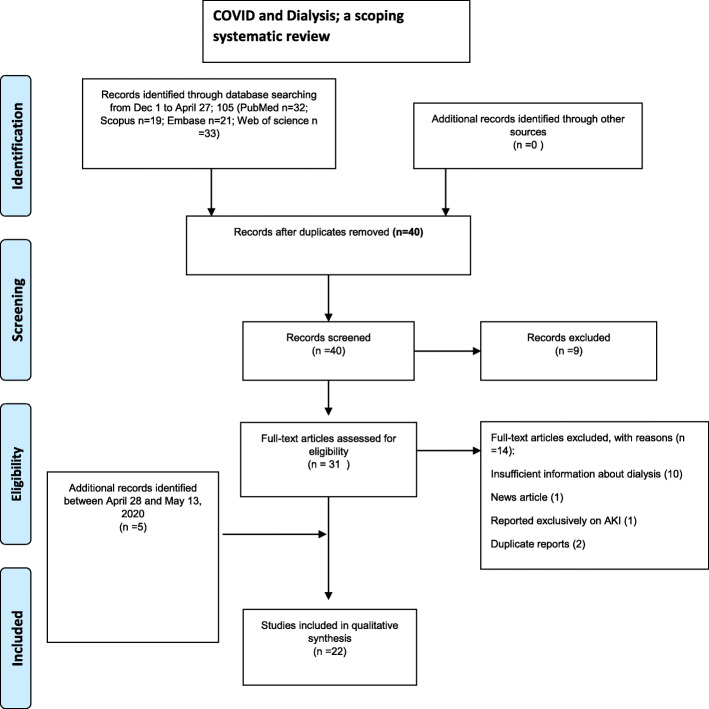
Preferred reporting items for systematic reviews and meta-analyses (PRISMA) flow diagram of the study

We performed a multi-step search strategy (Fig. 1). A limited preliminary search was done in Google Scholar, Scopus, and Pubmed to identify papers on this topic. We analyzed the keywords of the titles and abstracts to choose the most relevant and comprehensive terms. All authors discussed the terms and finalized the search strategy. Based on the output of the first step, two authors systematically searched the literature in the English language in Pubmed, Scopus, Embase, and Web of Science published from December 1, 2019, to May 13, 2020. We used the following keywords in our search (“Dialysis” OR “RRT” OR “Renal replacement therapy”) AND (“SARS-COV-2” or “COVID-19” OR “novel coronavirus” OR “2019-nCov”) (Table 1). There were a total of 105 papers in our first search from December 12,019 to April 27,2020 (PubMed *n* = 32; Scopus *n* = 19; Embase *n* = 21; Web of science *n* = 33). After removing the duplicate articles, the final result was 31 papers. Two authors separately screened the title and abstracts of the documents and ruled out irrelevant articles. We obtained a full report of the papers that met our inclusion criteria, screened the full texts, and excluded 11 articles that did not meet our standards: editorials, commentaries, and prospective with insufficient information. We searched databases another time from April 28 to May 13, 2020, that resulted in 82 papers, of which finally five was included in our study; two articles with duplicate reports were excluded. The final output was 22 papers. Authors were not blinded, and they independently participated in all phases of the project, including initial screening, and checking for eligibility.

**Table 1:** Search strategy

databases	Search strategy
Pubmed, Embase	[Title/Abstract] (“Dialysis” OR “RRT” OR “Renal replacement therapy”) AND (“SARS-COV-2” OR “COVID-19” OR “novel coronavirus” OR “2019-nCov”)
Web of sciences, Scopus	[Title/Abstract/Keywords] (“Dialysis” OR “RRT” OR “Renal replacement therapy”) AND (“SARS-COV-2” or “COVID-19” OR “novel coronavirus” OR “2019-nCov”)

### Inclusion criteria

We included both quantitative and qualitative studies if they met the conditions listed below:

1. The study assesses dialysis patient conditions during the COVID-19 pandemic
2. The study population was dialysis patients
3. The study was published in English
4. The study was published in a peer-reviewed journal.
5. The study was published between December 1, 2019, to May 13, 2020.

### Exclusion criteria

1. Not related to COVID-19
2. Not related to dialysis
3. Exclusively reporting on the renal complication of COVID-19
4. Studies about extracorporeal membrane oxygenation for severe pneumonia
5. Studies indirectly mentioning RRT
6. Studies reporting exclusively on acute kidney injury (AKI)

### Data extraction

The data were extracted from the included studies following this list:

1. Publication data (author, year of publication, the country of the first author).
2. Study type (perspective, review, guideline, case report/series, editorial, comment)
3. Patient population (ESRD/AKI population).
4. Results about recommendations for hemodialysis units, resource management, frequency of dialysis.
5. Limitations/biases.

Analysis of information needs:

We conducted a descriptive analysis of the characteristics of the included articles and performed a narrative synthesis of the results. Characteristics included: type of article, topic of article, patient population, publication year, country of publication, and source. We categorized the types of articles into perspectives, editorial, case reports, case series, guidelines, reviews, and comments. We conducted this scoping review in accordance with the PRISMA-ScR Checklist.

## Results

We retrieved 188 articles, and after removing the duplicate and unrelated records, 36 full-text articles were assessed for eligibility. Twelve articles were deemed ineligible (insufficient information about dialysis: *n = 10*; news report: *n = 1*; reported exclusively on AKI: *n = 1*). Twenty-two articles were included in the final analysis.

The article type varied vastly, which we broadly categorized into seven types (Table 2). Perspectives (*n = 9*; 40%), editorials (*n = 4*; 18%), and case series (*n = 5*; 22.7%) were the most common types of articles. Fifteen (68%) articles focused on the topic of recommendation for hemodialysis units. The patient populations studied were adults with end-stage renal disease (ESRD) in 20 articles and children with ESRD in 2 articles. All of the articles were published in the year 2020. We have categorized the recommendations into three categories, based on the intended stakeholder: 1) patients, 2) dialysis staff, and 3) hemodialysis facilities. For each one of the stakeholder groups, we have highlighted the recurring themes identified in the articles.

**Table 2:** Characteristics of the COVID-19 and dialysis articles Included in the scoping review, December 1, 2019, to May 13, 2020 (*n* = 22)

	Number
Type of article	
Perspective [17–25]	9
Editorial [14, 26–28]	4
Case series [29–33]	5
Case reports [34, 35]	2
Guideline [36]	1
Review [15]	1
Topic	
Recommendation for hemodialysis units [14, 15, 17–20, 22–25, 27, 28, 33, 34, 36]	15
Management / Implementation [29, 30, 32, 35]	4
Frequency of dialysis [21, 26]	2
Epidemiology [31]	1
Patient population	
End stage renal disease (adult) [14, 15, 17–32, 34, 35]	20
End stage renal disease (pediatrics) [33, 36]	2

### Patient-related recommendations

- *Education*
  - ○ Educate patients about COVID-19 symptoms and prevention, including hand hygiene, the use of Personal Protective Equipment (PPE), coughing etiquette, and discarding contaminated items [17–20, 29, 36].
  - ○ Provide guidance about nutrition and mental support [36].
  - ○ Encourage patients to call the dialysis unit if they are symptomatic [37].
  - ○ Educate patients about the risk of unnecessary talking or eating during dialysis [36].
- *Screening:*
  - ○ Patients should call the dialysis unit prior to their shift to respond to screening questions (symptoms, potential exposure, travel) [20, 31, 37].
  - ○ If a phone call is not possible, all patients should be screened upon entering the dialysis unit [14, 17–20, 22, 34]. As the epidemic evolves, the screening questions should be changed [22].
- *Temperature check:*
  - ○ The typical recommendation is to check the patients’ temperature before entering the dialysis unit [14, 17–19, 22, 29, 34, 36, 37], and also during dialysis [14].
  - ○ The red flag temperature ranges between 37.3 and 38 degrees Celsius (°C) [14, 17].
- *Personal Protective Equipment (PPE):*
  - ○ Wear a face mask when entering the unit, and throughout dialysis, or if symptomatic [14, 17, 18, 22, 29, 31, 32, 34, 36].
  - ○ Change shoes and clothes upon entering the unit [29].
- *Observe social distancing:*
  - ○ Stay at home while off dialysis and on non-dialysis days, and avoid unnecessary travel [15].
  - ○ Use private transportation to and from dialysis facilities, avoid public transportation [15, 31].
  - ○ Arrive at the time of dialysis to avoid exposure in the waiting area [36].
  - ○ Wait in the personal vehicle until the time of dialysis [20].
  - ○ Seek virtual medical care when possible [36].
  - ○ Avoid contact with children, if possible [15].
- *Accompanying persons:*
  - ○ The number of accompanying persons should be limited [36].
  - ○ Accompanying persons should receive education and information about COVID-19, its prevention, and the use of PPE [6, 36].
  - ○ Accompanying persons should wear mask, answer screening questions [6, 36], and their temperature should be checked before entering the dialysis unit [15, 36].
  - ○ Close contacts of patients with suspected or confirmed COVID-19 should be monitored for 14 days after the most recent contact [36] and should be tested for COVID-19 [18].
  - ○ Contact tracing, assessment of exposure, and optimal symptom-based testing strategies are essential to prevent outbreaks of SARS-CoV-2 [33].
- *Suspected or COVID-positive patients:*
  - ○ Patients with symptoms of fever, upper respiratory illness, cough, or conjunctivitis should be referred for testing and evaluation before allowing entry to the dialysis unit [18, 34, 36].
  - ○ Symptomatic patients who are awaiting test results, and those with positive contacts, should be treated as positive and should be admitted [20] for dialysis if dialysis cannot be postponed [18].
  - ○ The choice of providing either intermittent hemodialysis or CRRT should be based on a patient’s clinical status and the facility’s resources [38]. It is noteworthy that during the exponential surge of new cases of COVID-19 requiring RRT, there was a significant shortage of dialysis supplies, including CRRT solutions, particularly in the United States [39].
  - ○ For infection control purposes, healthcare personnel exposed to patients with suspected or confirmed COVID-19 should be limited to those essential for their care [38].
  - ○ Patients with a positive test should be dialyzed in the isolation room [18, 20, 30].
    - ▪ If no isolation room is available, they should be cohorted in a designated shift, a designated facility, or a corner or end of row chair/bed [20].
    - ▪ The duration of isolation precautions should be determined on a case-by-case basis in conjunction with local, state, and federal health authorities until the information is available regarding viral shedding after clinical improvement [15].
    - ▪ Single-center reports have recommended that patients with a positive test should be tested periodically [27], and should not return to the regular dialysis unit until they have had two negative tests (24-h apart) [36]. However, the Centers for Disease Control and Prevention (CDC) recommends that a symptom-based strategy should be used to guide the decision to discontinue transmission-based precautions, except among patients who are severly immunocompromised [40].

### Staff-related recommendations

- *Education*: Staff should receive education and training regarding the COVID-19 epidemic, epidemic prevention, use of PPE, respiratory hygiene, coughing etiquette, how to dispose contaminated items, and how to take nasopharynx swabs for COVID-19 [6, 19, 36].
- *Screening*:
  - ○ Staff should self-monitor symptoms, including twice-daily temperature check [17, 36], and should report any temperature of ≥37.3 °C [6] to their supervisor.
  - ○ Staff should inform the supervisor if they, or their family members, develop symptoms suggestive of COVID-19 infection, or if suspected or confirmed contact or travel to an epidemic area [36].
    - ▪ In the event of a history of close contact with a COVID-positive person, staff should only return to work if testing by SARS-CoV-2 nucleic acid is negative [6].
    - ▪ If there is any suspected history of exposure, staff should self-quarantine for at least 14 days [22, 36].
  - ○ Staff should undergo “at gate” infectious risk assessment (travel, occupation, contact, and clustering), and temperature check [19] [22].
  - ○ All dialysis staff should be tested periodically [27].
- *PPE:*
  - ○ All personnel involved in the direct care of patients suspected or affected by COVID-19 must use full protection PPE including air-purifying respirators, protective glasses/goggles, disposable gloves, waterproof disposable gown, head covering, for the entire duration of dialyzing COVID-19 positive patients [14, 15, 17, 18, 27, 29, 31, 34, 36].
  - ○ During the pandemic, and when dialyzing non-COVID positive patients, dialysis staff should wear masks, goggles, face screens, head covering, protective isolation gown, gloves, and shoe covers for connecting patients, blood draws, injections, or any other intervention with the risk of contact with the patient’s blood and body fluids [14, 17, 20, 22, 30, 36].

### Dialysis facility-related recommendations

- *Maintain physical distancing:*
  - ○ Observe general social distancing recommendations [14]
  - ○ Reduce group rounds and activities and transition to phone or online activities. When gathering is necessary, wear protective equipment [17] or hold meeting outdoors, if possible [29].
  - ○ Distance between dialysis chairs should be at least 6 ft [20, 29].
  - ○ Adapt waiting rooms for social distancing [14].
  - ○ Encourage stable patients to wait outside or in their personal vehicle [15].
  - ○ As an added measure to social distancing and to create an additional barrier, curtains should be drawn around the patient during treatment [36].
- *Isolate and cohort:*
  - ○ Notify direct patient care staff of the presence of a symptomatic patient [37] [37].
  - ○ Every effort should be made to keep COVID-positive patients out of the non-designated dialysis units [18] and to separate suspected and confirmed cases [15].
  - ○ Isolation dialysis units should be established in COVID-19–designated hospitals to manage COVID-19–positive patients on hemodialysis by dedicated staff to avoid cross-infection [6, 15].
  - ○ In non-COVID-19 designated hospitals, designated areas for dialyzing suspected and confirmed COVID-positive patients should be identified [15, 27, 30, 31, 34].
  - ○ Suspected or confirmed COVID-19 patients and a designated healthcare team should be cohorted in the same section of the unit and/or on the same shift (e.g., consider the last shift of the day). Only the assigned healthcare team should enter the isolation room/cohort area [6, 15, 19, 36].
  - ○ A separate transportation system devoted solely to COVID-19 positive patients should be established [29].
  - ○ Patients with shared transportation should be dialyzed in the same section of the dialysis unit treated with the same nurses to allow for tracing of contacts of those who become positive [14].
  - ○ COVID-positive dialysis patients in remote areas without the possibility of isolation, infectious diseases specialist, and intensive care units should be transferred to centralized hospitals [18].
  - ○ Resources from different dialysis organizations should be coordinated to enable easy cohorting and management of patients with COVID-19.
- *Avoid cross-contamination:*
  - ○ Supplies and equipment (including dialysis machines) dedicated to the isolation area, should remain in the isolation area between dialysis sessions [14, 36], and should be used only for COVID-19 patients [34].
  - ○ The units should dedicate a space for gowning and de-gowning of staff [30].
  - ○ If possible, each patient should use the same dialysis machine at every treatment [36], and patients should be discouraged from unnecessary change of dialysis units [6, 36].
  - ○ The units should maintain a relatively fixed medical staff to patients group [22].
- *Preserve resources:*
  - ○ Avoid staffing shortage by efficient use of workforce:
    - ▪ Recruit retired nurses and diversify training of new nurses [18].
  - ○ Encourage judicious use of PPE [19], keep track of PPE inventory, and prioritize and preserve PPE .
  - ○ Consider extended use of eye and face protection and consider recycling of PPE .
  - ○ To avoid unnecessary use of hospital beds, judicious recommendations for admissions are critical [14].
- *Maintain staff morale:*
  - ○ Foster an environment of transparency, where staff and patients feel valued and safe [19].
  - ○ Supervisors should maintain visible presence and leadership, communicating with patients and staff, sharing current-state information and plan, and seeking input [14, 19]. Recognize everyday heroes who inspire others, who promote optimism, and who are doing things right [24].
  - ○ Address the mental and physical well-being of staff [36].
  - ○ Ensure adequate rest to avoid accidental contamination due to exhaustion [36].
- *Considerations for home dialysis* [15, 18, 25, 35, 36]:
  - ○ For patients who are eligible, home dialysis (peritoneal dialysis [PD] or home hemodialysis) may be an attractive alternative, as it combines dialysis with social distancing and elimination of transportation needs [35].
  - ○ Special precautions are needed for patients on PD:
    - ▪ Air the room at least twice daily for 30 min each time, by opening windows and doors when PD is not running [36].
    - ▪ Sweep and clean the floor before the PD treatment once a day, and then carry out ultraviolet disinfection [25, 36].
    - ▪ Disinfect the procedure table and the automated PD machine (if applicable), before and after each PD treatment [36].
    - ▪ Disinfect the drainage fluid [36].
  - ○ PD and home hemodialysis patients should use telehealth rather than in-person visits to avoid unnecessary travel and clinic/hospital visits [15, 18, 36].
  - ○ For home dialysis patients, consider the option of visit by laboratory services to draw blood for monthly tests [28].
- *Role of CRRT or variations of maintenance hemodialysis for ESRD patients admitted to the hospital during the pandemic:*
  - ○ Critically ill maintenance hemodialysis patients with suspected or confirmed COVID-19 should undergo CRRT in cohorted units. This decreases dialysis nurse exposure to patients with COVID-19 [6, 36].
  - ○ Other options, in addition to CRRT, include sustained, low-efficiency dialysis (SLED), prolonged intermittent renal replacement therapy, or high-volume hemofiltration (6 L/hr), with adsorbent membranes to remove inflammatory cytokines and endotoxins [29].
  - ○ Urgent start peritoneal dialysis is an option when hemodialysis supply is low [23].
  - ○ Opinions differ concerning decreasing the frequency of hemodialysis during the pandemic. While some see twice-weekly hemodialysis only as an option of last resort [26], others cite the potential benefits as less exposure to COVID-19 for the patients and staff; reduction in dialysis staff work, a greater spacing of patients; reduced transportation needs; and conservation of PPE [21].
- *Other recommendations:*
  - ○ Place clear signs with consistent messages [19] to direct symptomatic or exposed patients to a designated screening location in an appropriate space, where the triage protocol can be implemented.
  - ○ Maintain appropriate ventilation in all the rooms, including the waiting room [15].
  - ○ Disinfect all the rooms and machines at the start of each shift [29].
  - ○ Maintain a checklist for implementation of disinfection of areas previously not routinely addressed [19].
  - ○ Discard all dialyzers and blood tubing as infectious waste [34].

## Conclusions

This scoping review provides a synopsis of articles relating to COVID-19 and maintenance outpatient hemodialysis. Most of the articles on COVID-19 and dialysis were focused on single-center experience and recommendations and were surprisingly almost non-existent in some fields, such as clinical research. Given the implications of COVID-19 in care of patients with end-stage renal disease, and also the need for renal replacement therapy among patients who develop acute kidney injury in the setting of COVID-19 infection, we anticipated a more significant number of articles discussing the clinical care and outcomes. The majority of articles were perspectives and editorials focusing on recommendations for adults with end-stage renal disease on hemodialysis. While we identified many overlapping recommendations, there were also several unique ideas: fostering an environment of transparency, with visible leadership and clear and transparent communication [14, 19], and recognizing staff who inspire others [24]; addressing the mental and physical well-being of staff, and ensuring adequate rest [36]; changing the screening questions, as the pandemic evolves [22]; shortening dialysis time or frequency, or decreasing dialysate flow rate to preserve resources [21]; dialyzing patients with shared transportation in the same section of the outpatient dialysis unit treated with the same nurses to allow for contact tracing [14]; re-checking temperature with mercury or ear thermometer if T > 37.3 °C [17]; changing shoes and clothes upon entering the unit [29], and considering home visits for lab draws [28]. We conclude that, given the rapidly evolving nature of the pandemic, while there were no pre-existing guidelines, clinical centers developed best practices. There is paucity of data regarding the outcomes resulting from implementation of recommendations and comparison of recommendations. Many of these practices have formed the basis for policies and guidelines that will guide future prevention of infection and management of patients with ESRD and COVID-19. This review underscores the need for extensive sharing of information between centers, large and small and within the healthcare community, as well as systematic reviews and meta-analyses which will form the basis for evidence-based guidelines.

## Data Availability

All data generated or analysed during this study are included in this published article.

## Abbreviations

AKI: Acute Kidney Injury
COVID-19: Coronavirus Disease-19
CRRT: Continuous Renal Replacement Therapy
CT: Computerized Tomography
ESRD: End Stage Renal Disease
ICU: Intensive Care Unit
MERS: Middle East Respiratory Syndrome
PD: Peritoneal Dialysis
PPE: Personal Protective Equipment
PRISMA: Preferred Reporting Items for Systematic Reviews
SARS: Severe Acute Respiratory Syndrome
WHO: World Health Organization

## References

[1] Lu H, Stratton CW, Tang YW. Outbreak of pneumonia of unknown etiology in Wuhan, China: The mystery and the miracle. J Med Virol. 2020;92(4):401-2. 10.1002/jmv.25678 PMC7166628 31950516

[2] Zhu N, Zhang D, Wang W, Li X, Yang B, Song J (2020). A novel coronavirus from patients with pneumonia in China, 2019. N Engl J Med.

[3] Xu J, Zhao S, Teng T, Abdalla AE, Zhu W, Xie L (2020). Systematic comparison of two animal-to-human transmitted human coronaviruses: SARS-CoV-2 and SARS-CoV. Viruses..

[4] Backer JA, Klinkenberg D, Wallinga J (2020). Incubation period of 2019 novel coronavirus (2019-nCoV) infections among travellers from Wuhan, China, 20–28 January 2020. Eurosurveillance..

[5] Zhou F, Yu T, Du R, Fan G, Liu Y, Liu Z (2020). Clinical course and risk factors for mortality of adult inpatients with COVID-19 in Wuhan, China: a retrospective cohort study. Lancet (London, England).

[6] Naicker S, Yang CW, Hwang SJ, Liu BC, Chen JH, Jha V (2020). The novel coronavirus 2019 epidemic and kidneys. Kidney Int.

[7] Zheng Y-Y, Ma Y-T, Zhang J-Y, Xie X (2020). COVID-19 and the cardiovascular system. Nat Rev Cardiol.

[8] Gu J, Han B, Wang J (2020). COVID-19: gastrointestinal manifestations and potential fecal-Oral transmission. Gastroenterology..

[9] Asadi-Pooya AA, Simani L (2020). Central nervous system manifestations of COVID-19: a systematic review. J Neurol Sci.

[10] Medjeral-Thomas NR, Thomson T, Ashby D, Muthusamy A, Nevin M, Duncan N, Loucaidou M. Cohort Study of Outpatient Hemodialysis Management Strategies for COVID-19 in North-West London. Kidney Int Rep. 2020;5(11):2055–65. 10.1016/j.ekir.2020.08.022 PMC7446656 32864514

[11] Cho JH, Kang SH. Hemodialysis with Cohort Isolation to Prevent Secondary Transmission during a COVID-19 Outbreak in Korea. 2020;31(7):1398–408. 10.1681/ASN.2020040461 PMC7351011 32482688

[12] Taghrir MH, Akbarialiabad H, Marzaleh MA (2020). Efficacy of mass quarantine as leverage of health system governance during COVID-19 outbreak: a mini policy review. Arch Iran Med.

[13] Rincón A, Moreso F, López-Herradón A, Fernández-Robres MA, Cidraque I, Nin J (2020). The keys to control a COVID-19 outbreak in a haemodialysis unit. Clin Kidney J.

[14] Meijers B, Messa P, Ronco C (2020). Safeguarding the maintenance hemodialysis patient population during the coronavirus disease 19 pandemic. Blood Purif.

[15] Basile C, Combe C, Pizzarelli F, Covic A, Davenport A, Kanbay M (2020). Recommendations for the prevention, mitigation and containment of the emerging SARS-CoV-2 (COVID-19) pandemic in haemodialysis centres. Nephrol Dial Transplant.

[16] Allen M, Bhanji A, Willemsen J, Dudfield S, Logan S, Monks T. A simulation modelling toolkit for organising outpatient dialysis services during the COVID-19 pandemic. PLoS One. 2020;15(8):e0237628. 10.1371/journal.pone.0237628 PMC7425906 32790773

[17] Li J, Xu G (2020). Lessons from the experience in Wuhan to reduce risk of COVID-19 infection in patients undergoing long-term hemodialysis. Clin J Am Soc Nephrol.

[18] Rombolà G, Heidempergher M, Pedrini L, Farina M, Aucella F, Messa P (2020). Practical indications for the prevention and management of SARS-CoV-2 in ambulatory dialysis patients: lessons from the first phase of the epidemics in Lombardy. J Nephrol.

[19] Watnick S, McNamara E (2020). On the frontline of the COVID-19 outbreak: keeping patients on long-term Dialysis safe. Clin J Am Soc Nephrol.

[20] Kliger AS, Silberzweig J (2020). Mitigating risk of COVID-19 in Dialysis facilities. Clin J Am Soc Nephrol.

[21] Meyer TW, Hostetter TH, Watnick S (2020). Twice-weekly hemodialysis is an option for many patients in times of Dialysis unit stress. J Am Soc Nephrol.

[22] Lee J-J, Lin C-Y, Chiu Y-W, Hwang S-J (2020). Take proactive measures for the pandemic COVID-19 infection in the dialysis facilities. J Formos Med Assoc.

[23] Goldfarb DS, Benstein JA, Zhdanova O, Hammer E, Block CA, Caplin NJ (2020). Impending shortages of kidney replacement therapy for COVID-19 patients. Clin J Am Soc Nephrol.

[24] Harwood L (2020). Pandemic uncertainty: considerations for nephrology nurses. Nephrol Nurs J.

[25] Lai XL, Wang HY, Guo ZY (2020). Recommendations for prevention and management of COVID-19 in peritoneal dialysis patients. Chronic Dis Transl Med.

[26] Mehrotra R (2020). Counterpoint: twice-weekly hemodialysis should be an approach of last resort even in times of Dialysis unit stress. J Am Soc Nephrol.

[27] Rombolà G, Brunini F (2020). COVID-19 and dialysis: why we should be worried. J Nephrol.

[28] El Shamy O, Sharma S, Winston J, Uribarri J (2020). Peritoneal Dialysis during the coronavirus Disease-2019 (COVID-19) pandemic: acute inpatient and maintenance outpatient experiences. Kidney Med.

[29] Scarpioni R, Manini A, Valsania T, De Amicis S, Albertazzi V, Melfa L, Ricardi M, Rocca C. Covid-19 and its impact on nephropathic patients: the experience at Ospedale "Guglielmo da Saliceto" in Piacenza. G Ital Nefrol. 2020;37(2):2020-vol2. 32281756

[30] Farina M, Barbisoni F, Bertacchini S, Borettaz I, Bucci R, Maggio M, Ronga C. An account of the first hours of the Covid-19 epidemic at the Nephrology Unit in Lodi (Lombardy). G Ital Nefrol. 2020;37(2):2020-vol2. 32281755

[31] Manganaro M, Baldovino S (2020). First considerations on the SARS-CoV-2 epidemic in the Dialysis units of Piedmont and Aosta Valley, Northern Italy. J Nephrol.

[32] Alberici F, Delbarba E, Manenti C, Econimo L, Valerio F, Pola A (2020). Management of patients on Dialysis and with kidney transplant during SARS-COV-2 (COVID-19) pandemic in Brescia, Italy. Kidney Int Rep.

[33] Schwierzeck V, König JC, Kühn J, Mellmann A, Correa-Martínez CL, Omran H, Konrad M, Kaiser T, Kampmeier S. First reported nosocomial outbreak of severe acute respiratory syndrome coronavirus 2 (SARS-CoV-2) in a pediatric dialysis unit. Clin Infect Dis. 2020. p. ciaa491. 10.1093/cid/ciaa491 PMC7197625 33501962

[34] Tang B, Li S, Xiong Y, Tian M, Yu J, Xu L (2020). COVID-19 pneumonia in a hemodialysis patient. Kidney Med.

[35] Ferrey AJ, Choi G, Hanna RM, Chang Y, Tantisattamo E, Ivaturi K (2020). A case of novel coronavirus disease 19 in a chronic hemodialysis patient presenting with gastroenteritis and developing severe pulmonary disease. Am J Nephrol.

[36] Shen Q, Wang M, Che R, Li Q, Zhou J, Wang F (2020). Consensus recommendations for the care of children receiving chronic dialysis in association with the COVID-19 epidemic. Pediatr Nephrol.

[37] CDC (2020). Screening Dialysis Patients for COVID-19.

[38] CDC (2020). Considerations for Providing Hemodialysis to Patients with Suspected or Confirmed COVID-19 in Acute Care Settings.

[39] Mahase E (2020). Covid-19: increasing demand for dialysis sparks fears of supply shortage. BMJ..

[40] CDC (2020). Testing Guidelines for Outpatient Dialysis Facilities.

